# Complete chloroplast genome of *Gracilaria firma* (Gracilariaceae, Rhodophyta), with discussion on the use of chloroplast phylogenomics in the subclass Rhodymeniophycidae

**DOI:** 10.1186/s12864-016-3453-0

**Published:** 2017-01-06

**Authors:** Poh-Kheng Ng, Showe-Mei Lin, Phaik-Eem Lim, Li-Chia Liu, Chien-Ming Chen, Tun-Wen Pai

**Affiliations:** 1 Institute of Marine Biology, National Taiwan Ocean University, Keelung, 20244 Taiwan; 2 Institute of Ocean and Earth Sciences, University of Malaya, Kuala Lumpur, 50603 Malaysia; 3 Department of Computer Science and Engineering, National Taiwan Ocean University, Keelung, 20244 Taiwan

**Keywords:** Chloroplast genome, *Gracilaria firma*, Gracilariaceae, Conserved synteny, Phylogenomic analyses, Red algal plasmid remnants

## Abstract

**Background:**

The chloroplast genome of *Gracilaria firma* was sequenced in view of its role as an economically important marine crop with wide industrial applications. To date, there are only 15 chloroplast genomes published for the Florideophyceae. Apart from presenting the complete chloroplast genome of *G. firma*, this study also assessed the utility of genome-scale data to address the phylogenetic relationships within the subclass Rhodymeniophycidae. The synteny and genome structure of the chloroplast genomes across the taxa of Eurhodophytina was also examined.

**Results:**

The chloroplast genome of *Gracilaria firma* maps as a circular molecule of 187,001 bp and contains 252 genes, which are distributed on both strands and consist of 35 RNA genes (3 rRNAs, 30 tRNAs, tmRNA and a ribonuclease P RNA component) and 217 protein-coding genes, including the unidentified open reading frames. The chloroplast genome of *G. firma* is by far the largest reported for Gracilariaceae, featuring a unique intergenic region of about 7000 bp with discontinuous vestiges of red algal plasmid DNA sequences interspersed between the *nblA* and *cpeB* genes. This chloroplast genome shows similar gene content and order to other Florideophycean taxa. Phylogenomic analyses based on the concatenated amino acid sequences of 146 protein-coding genes confirmed the monophyly of the classes Bangiophyceae and Florideophyceae with full nodal support. Relationships within the subclass Rhodymeniophycidae in Florideophyceae received moderate to strong nodal support, and the monotypic family of Gracilariales were resolved with maximum support.

**Conclusions:**

Chloroplast genomes hold substantial information that can be tapped for resolving the phylogenetic relationships of difficult regions in the Rhodymeniophycidae, which are perceived to have experienced rapid radiation and thus received low nodal support, as exemplified in this study. The present study shows that chloroplast genome of *G. firma* could serve as a key link to the full resolution of *Gracilaria sensu lato* complex and recognition of *Hydropuntia* as a genus distinct from *Gracilaria sensu stricto*.

**Electronic supplementary material:**

The online version of this article (doi:10.1186/s12864-016-3453-0) contains supplementary material, which is available to authorized users.

## Background

Rhodophyta is a monophyletic phylum currently divided into seven classes, including Bangiophyceae, Compsopogonophyceae, Cyanidiophyceae, Florideophyceae, Porphyridiophyceae, Rhodellophyceae and Stylonematophyceae [1, 2]. The Florideophyceae which accommodates more than 6700 species of red algae [3] has only chloroplast genomes of 15 species published to date. Despite the sporadic studies [4–6] on the phylogenetic relationships among the Florideophyceae inferred using chloroplast genome data, the whole chloroplast genome has been demonstrated to be a promising resource to resolve the red algal relationships, attributable to the conserved nature of the slowly-evolving genome [4]. In line with that, emergence of the next generation high-throughput sequencing technologies and the associated exponential decline in the cost of sequencing would open up the opportunity for more whole genome sequencing projects targeted to gain more novel insights into the evolutionary relationships within the red algal lineage.

*Gracilaria firma* Chang et Xia is an agar-producing seaweed distributed in the tropical and subtropical regions in the western Pacific [7–9]. It is an economically important marine crop that has been cultivated on commercial scale in several countries including Taiwan [10], Vietnam [11] and the Philippines [12]. The seaweed exemplifies superior growth and agar quality among other investigated Gracilarioid agarophytes [13]. Apart from serving as the source of agar which seeks versatile applications in food and pharmaceutical industries, *G. firma* is also harvested and made into local delicacies for human consumption [12]. Some regional abalone farms preferred using *G. firma* as the natural feed [10].

This study presents the complete chloroplast genome of *G. firma*, adding to the number of chloroplast genome available for the genus *Gracilaria*, one of the largest red algal genera which encompassed more than a hundred species [3] and has undergone numerous taxonomic revisions with contradicting conclusions [14–16]. Analysis of the synteny and genome structure concurred that the red algal chloroplast genomes are very compact and conserved across the subphylum Eurhodophytina, with different orders exhibiting syntenies that discriminate lineages. Phylogenomic analyses including most of the recently available (as of the date of writing this manuscript in June 2016) taxa across eight orders of Florideophyceae were conducted. The relevance of chloroplast genomic data in addressing the phylogenetic relationships at different hierarchical levels in Rhodymeniophycidae was also discussed.

## Methods

### Taxon sampling and sequencing

The plant material of *G. firma* was procured from a cultivation farm operated in Kouhu Township, Yunlin County, Taiwan. Genomic DNA was extracted from fresh thallus of *G. firma* using the DNeasy Plant Mini Kit (Qiagen, Valencia, CA, USA) according to the manufacturer’s instruction and sent to a company for library prep and sequencing (ScienceVision Sdn Bhd, Selangor, Malaysia). A corresponding voucher specimen is deposited in the Herbarium of the National Taiwan Ocean University, Taiwan (NTOU) under the accession number NTOU-KH-5i2016-Gf. The library was prepared using a Nextera XT kit (Illumina, San Diego, CA, USA) and sequenced with 250 bp pair-end reads on the MiSeq sequencing platform (Illumina, San Diego, CA, USA).

### De novo *assembly and annotation of the* Gracilaria firma *chloroplast genome*

**Fig. 1:**
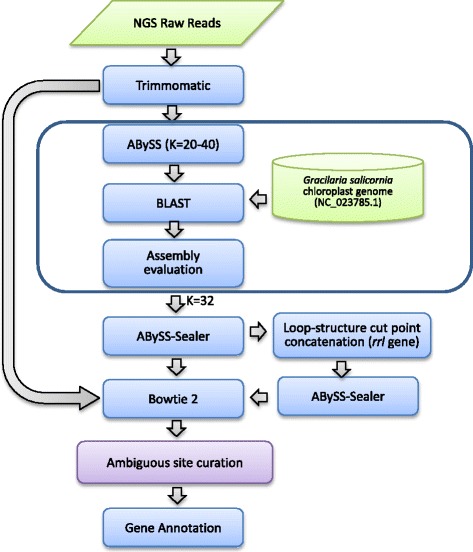
Graphical representation of the genome assembly pipeline for the chloroplast genome of *Gracilaria firma*

The genome assembly pipeline is shown in Fig. 1. The sequenced NGS raw reads were stored in Fastq format with a total of 7,962,495 read pairs, and trimmed using Trimmomatic v0.36 [17] with the following parameter settings: LEADING:15, TRAILING:15, SLIDINGWINDOW: 4:15 and MINLEN:36. A total of 7,490,913 read pairs (94.1%) survived filtering. The genome was first assembled using the ABySS *de novo* assembler v1.9.0 [18] with different k-mer parameters varying from 20 to 40, and the best assembly results were determined based on the information content estimates (total number of gene BLAST hits against the reference genome of *G. salicornia* (NC_023785)). A single contig of 186,354 bp with 150 mapped genes, assembled using the k-mer size of 32, was identified as the probable candidate of the complete chloroplast genome. Reads were also mapped back to the contig using Bowtie 2 [19] to validate the assembly. The contig was further refined using the Sealer tool [20] implemented in ABySS to verify the gaps represented by character *N* within the original ABySS output. Manual curation of the ambiguous sites reported from ABySS Sealer was performed via the visualization tool IGV v2.3 [21] based on the reference mapping results. As circularity was not inferred in the refined contig, the gap in the *rrl* gene was closed by applying the ABySS Sealer and manual ambiguous site curation to the concatenation of the end segments of the contig and a segment of *N* sequences (50 bases). The final genome was validated by mapping the reads against the refined contig, where 1,140,489 of the 14,981,826 filtered reads mapped to the chloroplast genome with a mean coverage of 996×. An independent genome assembly was also conducted with the IDBA-UD *de novo* assembler v1.0.9 [22] using mink = 35 and maxk = 100, coupled with the reference-guided ARC assembler v1.1.3 [23] using the reference genome of *G. tenuistipitata* (AY673996) to result in a single contig of 187,249 bp. One of the self-similar ends encoding the *groEL* gene was manually trimmed to yield a contig representative of the circular chloroplast genome of *G. firma*. Both assembly approaches yielded single contig with identical sequences.

A combination of automated pipelines and manual verification was used to annotate the chloroplast genome of *G. firma*. Initial gene calling was accomplished using DOGMA [24] with a 60% cutoff for protein-coding genes, 80% for RNAs, and an *e*-value cut-off of 1e-5 for BLAST hits. The rRNA genes were determined using RNAmmer 1.2 server [25] with the ‘kingdom of input sequences’ selected as ‘Bacteria’. The tRNAs were identified using tRNAscan-SE v1.21 [26] with default parameters and the source identified as ‘Mito/Chloroplast’. Intron and tmRNA were searched using ARAGORN [27], while the ribonuclease P gene (*rnpB*) was detected using RNAweasel [28]. Open reading frames (ORFs) longer than 25 amino acids within the intergenic regions were searched using the NCBI’s ORF finder (https://www.ncbi.nlm.nih.gov/orffinder), with the genetic code option of ‘bacterial, archaeal and plant plastid’. BLASTp homology searches against all the non-redundant protein sequences from GenBank were used to determine the start and stop codon positions for each protein-coding gene, including those detected by DOGMA and the small or missing genes recovered from ORF finder. The circular genome map was generated using OGDraw [29]. The *G. firma* chloroplast genome sequence and annotation was deposited into GenBank under accession number KX601051.

### Synteny analyses on the red algal chloroplast genomes

Whole-genome alignments were generated using the progressiveMauve aligner implemented in Mauve v20150226 [30] under default settings. The synteny within the Gracilariales was assessed from the alignment of the chloroplast genomes of *G. firma* and related species from the same order, with which the genome sequences were reoriented to have the beginning of the *psaM* gene as the first position for alignment. In view of the variable genome structure as well as the lack of coherence in the designation of the first gene for chloroplast genomes across the Eurhodophytina, the synteny among the Florideophyceae and Bangiophyceae was assessed by visual inspection of the whole-genome alignments variously generated with the chloroplast genome of *G. firma* serving as the reference to detect the conserved segments of sequence free from any internal rearrangements, also known as the locally collinear blocks (LCBs).

### Phylogenomic analyses

Two datasets were assembled for the phylogenomic analyses based on the chloroplast genome data of 21 taxa of Rhodophyta, including the data newly obtained for *G. firma* (Table 1). Analyses on the protein-coding genes were performed with amino acid sequences to avoid phylogenetic artefacts caused by synonymous substitutions-induced convergent base composition [31]. The first dataset included 79 protein-coding genes present in all taxa, selected based on the recommendation in [4]: *acsF*, *apcA*, *apcB*, *apcD*, *apcE*, *apcF*, *atpA*, *atpB*, *atpF*, *atpG*, *atpI*, *carA*, *ccsA*, *chlI*, *clpC*, *cpcA*, *cpcB*, *cpcG*, *dnaK*, *ftrB*, *ftsH*, *groEL*, *infC*, *pdhA*, *pdhB*, *petA*, *petB*, *petF*, *petJ*, *preA*, *psaA*, *psaB*, *psaF*, *psbA*, *psbB*, *psbC*, *psbD*, *psbV*, *rbcR*, *rpl1*, *rpl2*, *rpl3*, *rpl4*, *rpl5*, *rpl6*, *rpl11*, *rpl12*, *rpl12*, *rpl16*, *rpl18*, *rpl19*, *rpl21*, *rpl22*, *rpl23*, *rpoB*, *rpoC1*, *rpoC2*, *rps2*, *rps3*, *rps4*, *rps5*, *rps7*, *rps10*, *rps11*, *rps12*, *rps13*, *rps14*, *secY*, *sufB*, *sufC*, *tatC*, *thiG*, *trpA*, *trxA*, *tsf*, *tufA*, *ycf4*, *ycf39* and *ycf65*. These genes are considered as the most promising candidate genes for phylogenetic, barcoding and population studies, as they demonstrated low non-synonymous substitution rates across distantly related red algae. In line with the aim of phylogenomics to reduce random or sampling error with the use of large dataset [32], a larger concatenated dataset representing the expansion of the first dataset with the protein-coding genes present in all examined taxa were also analyzed. The second dataset consisted of 146 protein-coding genes, including the following in addition to the first dataset: *acpP*, *atpD*, *atpE*, *atpH*, *cbbX*, *cemA*, *gltB*, *ilvB*, *ilvH*, *nblA*, *ntcA*, *petD*, *petG*, *psaD*, *psaE*, *psaI*, *psaJ*, *psaK*, *psaL*, *psaM*, *psbE*, *psbF*, *psbH*, *psbI*, *psbJ*, *psbK*, *psbL*, *psbN*, *psbT*, *psbW*, *psbX*, *psbY*, *psbZ*, *rbcL*, *rbcS*, *rpl13*, *rpl14*, *rpl20*, *rpl24*, *rpl27*, *rpl28*, *rpl29*, *rpl31*, *rpl32*, *rpl33*, *rpl34*, *rpl35*, *rpl36*, *rpoA*, *rpoZ*, *rps6*, *rps8*, *rps9*, *rps16*, *rps17*, *rps18*, *rps19*, *rps20*, *secA*, *tilS*, *trpG*, *ycf3*, *ycf19*, *ycf53*, *ycf54*, *ycf60* and *ycf80*.

**Table 1:** Red algal taxa analyzed in this study and their chloroplast genome composition

Species and GenBank accession number	Reference	Size (bp)	GC (%)	Protein-coding genes	tRNAs	rRNAs
Cyanidiophyceae						
*Cyanidioschyzon merolae* (AB002583)	[40]	149,987	37.6	207	31	3
*Cyanidium caldarium* (AF022186)	[55]	164,921	32.7	201	30	3
Porphyridiophycaeae						
*Porphyridium purpureum* (AP012987)	[56]	217,694	30.3	224	29	6
Bangiophyceae						
*Porphyra umbilicalis* (JQ408795)	[57]	189,933	32.9	209	37	6
*Pyropia haitanensis* (KC464603)	[44]	195,597	33.1	213	37	6
*Wildemania schizophylla* (KR028420)	[58]	193,008	34.4	211	34	4
*Bangia atropurpurea* (KR028420)	Unpublished	189,505	32.4	206	35	4
Florideophyceae						
Corallinophycidae						
*Calliarthron tuberculosum* (KC153978)	[4]	178,981	29.2	201	31	3
*Sporolithon durum* (KT266785)	[6]	191,465	29.3	202	30	3
Rhodymeniophycidae						
*Chondrus crispus* (HF562234)	[4]	180,086	28.7	204	30	3
*Gelidium elegans* (KT266786)	[6]	174,748	30.2	203	30	3
*Gelidium vagum* (KT266787)	[6]	179,853	29.9	201	30	3
*Gracilaria firma* (KX601051)	This study	187,001	28.1	219	30	3
*Gracilaria salicornia* (KF861575)	[41]	179,757	28.8	204	31	3
*Gracilaria chilensis* (KT266788)	[6]	185,637	29.3	203	30	3
*Gracilaria tenuistipitata* var. *liui* (AY673996)	[38]	183,885	29.2	207	31	3
*Gracilariopsis lemaneiformis* (KU179794)	[59]	182,505	27.4	204	31	3
*Grateloupia taiwanensis* (KC894740)	[42]	191,270	30.6	235	31	3
*Coeloseira compressa* (KU053957)	[60]	176,291	29.0	201	29	3
*Laurencia snackeyi* (LN833431)	[39]	174,935	30	200	29	3
*Vertebrata lanosa* (KP308097)	[43]	167,158	30	192	27	3

Amino acid sequences from each individual gene were aligned using MAFFT v7.222 [33] with the ‘L-INS-i’ strategy. Each individual alignment was subjected to trimming using the program Gblocks v0.91b available at http://phylogeny.lirmm.fr/phylo_cgi/one_task.cgi?task_type=gblocks [34] for the removal of ambiguous regions which were poorly aligned or contained gaps under the setting for a more stringent selection that does not allow many contiguous non-conserved positions. The alignments of each individual gene were variously concatenated using Bioedit v7.2.5 [35] to result in two datasets comprised of 20,033 and 27,205 positions each. Maximum likelihood (ML) tree search were implemented in PhyML v3.0 [36], based on the MtZoa + G + I + F and cpREV + G + I + F model automatically selected by the program for the 79-gene and 146-gene datasets respectively. Branch support for both datasets were evaluated using the SH-like approximate Likelihood Ratio Test (SH-aLRT) implemented in PhyML. Only the 146-gene dataset was subjected to bootstrap analysis with 1000 bootstrap replicates. Bayesian inference (BI) was conducted with MrBayes v3.2.6 [37], using the cpREV + G + I + F model on both datasets as the best-fitting MtZoa + G + I + F model deduced for the 79-gene dataset was not implemented in MrBayes, with two parallel independent runs, each of which consisted of one cold chain and three hot chains of Markov chain Monte Carlo iterations for one million generations. The trees were sampled every 100th generation. Convergence of the runs to the stationary distribution was determined by looking at the standard deviation of split frequencies (always less than 0.01) and by the convergence of the parameter values in the two independent runs. The first 25% of the total number of the trees were discarded as burn-in, and the remaining trees were used to calculate a 50% majority rule tree and to determine the posterior probabilities for all datasets. Members of the Cyanidiales, *Cyanidioschyzon merolae* and *Cyanidium caldarium* were designated as the outgroup taxa based on the global phylogenetic searches recovered for the red algae [2].

## Results and discussion

### *Characteristics of the chloroplast genome of* Gracilaria firma

**Fig. 2:**
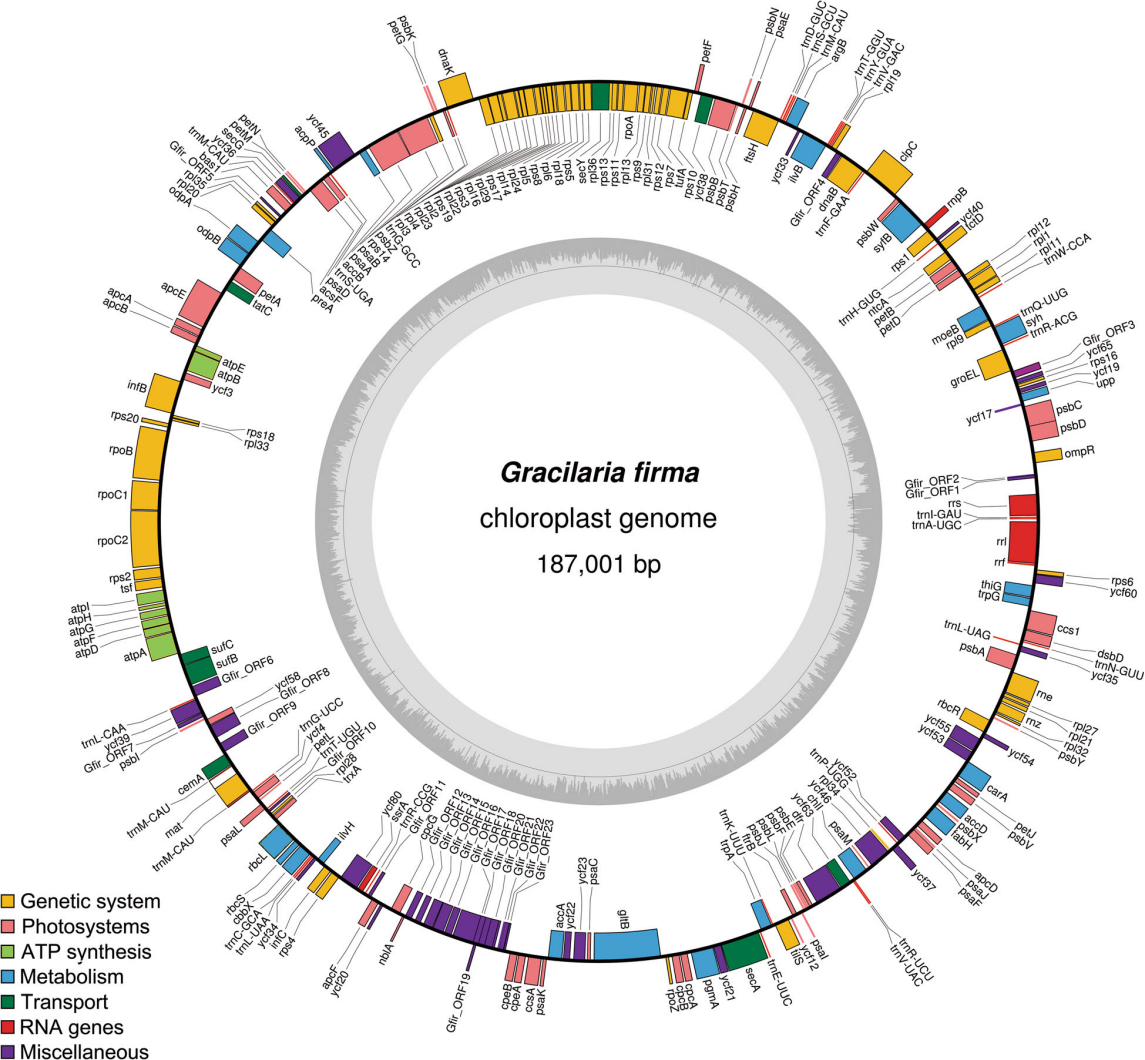
Chloroplast genome map of *Gracilaria firma.* Genes present inside the circle are transcribed in a clockwise direction whereas those outside counter clockwise. The bar graphs on the inner circle reveal GC content in dark grey with the 50% threshold line. The overall GC content of the genome is low, but there is an increase in GC content corresponding to the region encoding for rRNA genes. The annotated genes are color-coded according to the functional categories listed in the legend

**Fig. 3:**
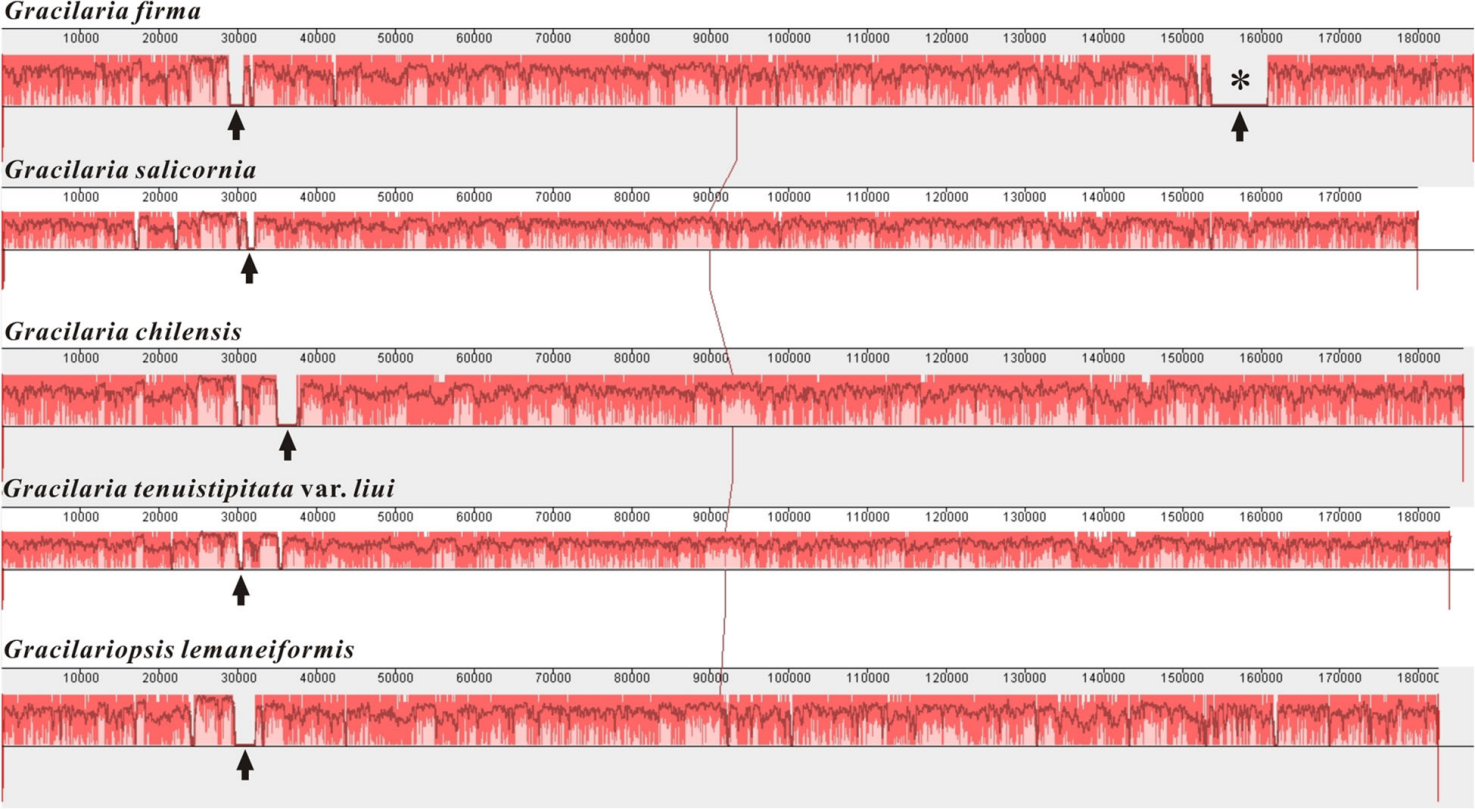
Whole chloroplast genome alignment of five taxa of Gracilariales. Linearized chloroplast genome of *Gracilaria firma* are aligned with previously published genome of *G. salicornia*, *G. chilensis*, *G. tenuistipitata* var. *liui* and *Gracilariopsis lemaneiformis*. All genomic sequences are designated to start with the *psaM* gene. Ruler above each genome represents nucleotide positions. The plot in red reflects the level of sequence similarity; the white region indicates element specific to a genome. Line links blocks with homology between two genomes. Arrow in all chloroplast genomes of Gracilariales represents the region of red algal plasmid integration in each genome. Asterisk represents the unique region of about 7000 bp containing plasmid-derived ORFs in the chloroplast genome of *G. firma*

The complete circular chloroplast genome of *G. firma* is 187,001 bp in length. *Gracilaria firma* has the largest chloroplast genome reported within the order Gracilariales thus far, with the chloroplast genomes of *G. salicornia*, *G. chilensis*, *G. tenuistipitata* var. *liui* and *Gracilariopsis lemaneiformis* each reporting the size of 179, 185, 183 and 183 kb*.* The genome has an overall GC content of 28.1% (Fig. 2). In contrast to the tRNA and rRNA genes which are richer in GC content (51.3 and 44.8% each on average), the protein-coding regions and intergenic spacers accounted for the generally low overall GC content (28.5 and 16.5% each on average). The genome size and base composition of *G. firma* is comparable to those reported for other chloroplast genomes in red algae and followed the typical AT-rich trend (Table 1). The standard start codon ATG was used in 90.8% of the ORFs of the chloroplast genome. Alternative start codons were also used to initiate translation, including ATC for *Gfir_ORF15*; GTG for *rps8, petM, rbcS, infC* and *psaF*; TTG for *ompR*, *ycf65*, *trxA*, *ycf20*, *Gfir_ORF13*; ATA for *tctD*, *rpl22*, *infB*, *Gfir_ORF11, Gfir_ORF17* and *Gfir_ORF23*; and ATT for *Gfir_ORF3*, *Gfir_21* and *chlI*. TAA was the most commonly used stop codon (72.4%), followed by TAG (15.7%) and TGA (12.0%). Despite the rather large variation in genome size, the chloroplast genome of *G. firma* demonstrated an extensive conservation of synteny to other members of Gracilariales, as evidenced by the single LCB resulted from the chloroplast genome alignment (Fig. 3).

Genetic information is densely packed in the chloroplast genome of *G. firma*, with the combined coding regions spanning 83.5% of the genomic sequence. The intergenic spacers were 122 bp long on average and overlapping ORFs between seven gene pairs were observed, including *psbC*-*psbD* (7 bp), *rpl24*-*rpl14* (1 bp), *rpl14*-*rps17* (4 bp), *rps17*-*rpl29* (4 bp), *rpl23*-*rpl4* (28 bp), *rps18*-*rpl33* (4 bp) and *atpF*-*atpD* (4 bp). Such overlapping is not unprecedented in the red algal chloroplast genome. For instance, the overlapping of *psbC*-*psbD* had been reported in *Gracilariopsis lemaneiformis*, *G. tenuistipitata* var. *liui* and *Laurencia snackeyi* [5, 38, 39]. *Cyanidioschyzon merolae* even showed up to 40% of gene overlapping in the chloroplast genome, including the *rps14*-*rps17* gene pair [40].

A total of 252 coding regions were predicted for the genome, including three rRNA genes (*rrs*, *rrl* and *rrf*), 30 tRNA genes, a sufficient set for the chloroplast protein synthesis machinery, one tmRNA (*ssra*, tag peptide: AKNNILNLSKQLVCV) gene, one ribonuclease P RNA component (*rnp*B), and 217 protein-coding genes including unidentified ORFs (Fig. 2, Table 2). As with the chloroplast genome of many other Florideophycean algae, that of *G. firma* featured only a single copy of rRNA operon composed of three rRNA genes and two tRNA genes (*trnI* and *trnA*) sandwiched between the *rrs* and *rrl* genes. Of the 30 tRNA in the chloroplast genome of *G. firma*, three encoded for arginine, two for valine, two for threonine, three for methionine, two for serine, two for glycine, and three for leucine. The other amino acids were encoded by single tRNA gene. One of the tRNA genes encoding methionine had a group II intron encoding a maturase. The presence of this group II intron in the *trnM* gene is not uncommon, as it is regarded as a signature characteristic of most florideophytes [4]. However, the intron can be easily overlooked during annotation [38]. Of the 217 protein-coding genes annotated on the *G. firma* chloroplast genome, 193 are widely conserved and named genes, including the conserved hypothetical genes. None of the protein-coding genes contains introns. Twenty three ORFs were annotated in the chloroplast genome of *G. firma*, of which 14 of them showed significant homology to the ORFs of red algal plasmids against BLASTp search with the threshold *e*-value of 1e-5 (Additional file 1), eight of them showed significant similarity with ORFs from other red algae (*e*-value <1e-5), and one of them showed remote similarity to the *pbsA* gene (*e*-value = 0.7). The gene content of the chloroplast genome of *G. firma* is very similar to that of other florideophyte chloroplast genomes, with suites of genes involved in photosynthesis, electron transport, translation, biosynthesis of amino acids, fatty acids, pigments and other cellular processes, apart from the lack of the *pbsA* gene which is also missing in *G. chilensis* [6].

**Table 2:** Functional classification of *Gracilaria firma* chloroplast genes

Classification	Genes
Genetic system	
Maintenance	*dnaB, rne, rnz, mat*
RNA polymerase	*rpoA, rpoB, rpoC1, rpoC2, rpoZ*
Transcription factors	*ompR, rbcR, tctD, ntcA*
Translation	*infB, infC, tsf, tufA*
Ribosomal proteins	*rpl1, rpl2, rpl3, rpl4, rpl5, rpl6, rpl9, rpl11, rpl12, rpl13, rpl14, rpl16, rpl18, rpl19, rpl20, rpl21, rpl22, rpl23, rpl24, rpl27, rpl28, rpl29, rpl31, rpl32, rpl33, rpl34, rpl35, rpl36, rps1, rps2, rps3, rps4, rps5, rps6, rps7, rps8, rps9, rps10, rps11, rps12, rps13, rps14, rps16, rps17, rps18, rps19, rps20*
tRNA processing	*tilS,*
Protein quality control	*clpC, dnaK, ftsH, groEL*
Photosystems	
Phycobilisomes	*apcA, apcB, apcD, apcE, apcF, cpcA, cpcB, cpcG, cpeA, cpeB, nblA, ycf58*
Photosystem I	*psaA, psaB, psaC, psaD, psaE, psaF, psaI, psaJ, psaK, psaL, psaM, ycf3, ycf4*
Photosystem II	*psbA, psbB, psbC, psbD, psbE, psbF, psbH, psbI, psbJ, psbK, psbL, psbN, psbT, psbV, psbW, psbX, psbY, psbZ, ycf12*
Cytochrome complex	*petA, petB, petD, petF, petG, petJ, petL, petM, petN, ccsA, ccs1*
Redox system	*acsF, bas1, ftrB, dsbD, trxA*
ATP synthesis	
ATP synthase	*atpA, atpB, atpD, atpE, atpF, atpG, atpH, atpI*
Metabolism	
Carbohydrates	*odpA, odpB, rbcL, rbcS, cbbX, pgmA*
Lipids	*accA, accB, accD, acpP, fabH*
Nucleotides	*carA, upp*
Amino acids	*argB, gltB, syh, ilvB, ilvH, trpA, trpG, syfB*
Cofactors	*chlI, moeB, preA, thiG*
Transport	
Transport	*cemA, secA, secG, secY, tatC, sufB, sufC, ycf38, ycf63*
Unknown	
Conserved ORFs	*dfr, ycf17, ycf19, ycf20, ycf21, ycf22, ycf23, ycf33, ycf34, ycf35, ycf36, ycf37, ycf39, ycf40, ycf45, ycf46, ycf52, ycf53, ycf54, ycf55, ycf60, ycf65, ycf80*
Unique ORFs	*Gfir_ORF1, Gfir_ORF2, Gfir_ORF3, …, Gfir_ORF23*
RNA genes	
rRNAs	*rrf, rrs, rrl*
tRNAs	*trnA, trnC, trnD, trnE, trnF, trnG, trnG, trnH, trnI, trnK, trnL, trnL, trnL, trnM, trnM, trnM, trnN, trnP, trnQ, trnR, trnR, trnR, trnS, trnS, trnT, trnT, trnV, trnV, trnW, trnY*
Miscellaneous RNAs	*ssrA, rnpB*

### *Red algal plasmid-derived regions in the chloroplast genome of* Gracilaria firma

**Fig. 4:**
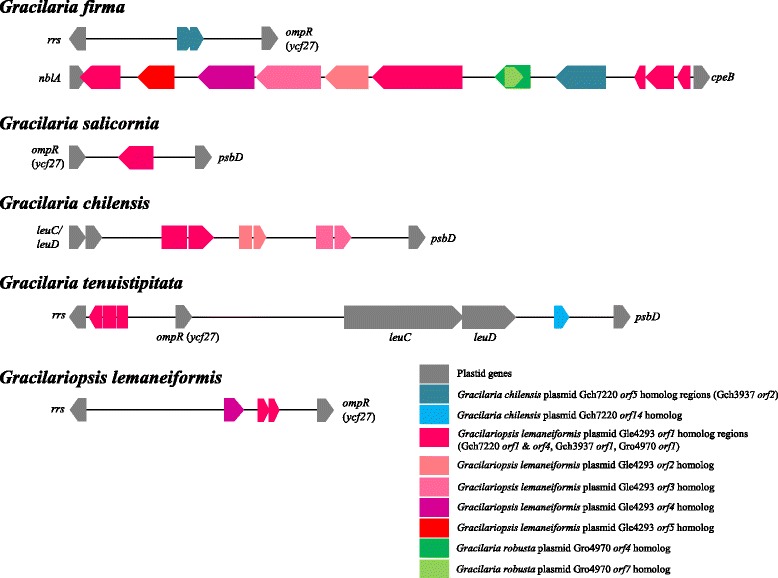
Schematic representation of the loci of the plasmid-related ORFs in the chloroplast genomes of Gracilariales. All taxa of Gracilariales harbor ORFs of red algal plasmid provenance in the intergenic regions within the *rrs-ompR-psbD* cluster. The chloroplast genome of *G. firma* consists of a unique stretch of 12 plasmid-related ORFs in the intergenic region between the *nblA* and *cpeB* genes. The position of the plasmid-related ORFs in the chloroplast genome of *G. salicornia*, *G. chilensis* and *G. tenuistipitata* var. *liui* is adapted from [5, 6]

A total of 14 ORFs homologous to red algal plasmids (*Gfir_ORF1*, *Gfir_ORF2*, *Gfir_ORF12*, *Gfir_ORF13*, *Gfir_ORF14*, *Gfir_ORF15*, *Gfir_ORF16*, *Gfir_ORF17*, *Gfir_ORF18*, *Gfir_ORF19*, *Gfir_ORF20*, *Gfir_ORF21*, *Gfir_ORF22* and *Gfir_ORF23*) are interspersed in the *rrs*-*ompR* and *nblA-cpeB* intergenic regions of the *G. firma* chloroplast genome, with *Gfir_ORF1* and *Gfir_ORF2* found in the former spacer, and the remaining in the latter (Fig. 4). These ORFs showed moderate to strong similarity to ORFs found in the plasmids of *G. chilensis* (Gch7220 and Gch3937), *G. robusta* (Gro4970) and *Gp. lemaneiformis* (Gle4293) (Additional file 1). ORF5 in Gch7220 is almost similar to ORF2 in Gch3937 with 99% amino acid identity. ORF1 in Gle4293 is similar to ORF1 and ORF4 in Gch7220, ORF1 in Gch3937 and ORF1 in Gro4970 with 34-38% amino acid identity, all of which constituting part of the protein family DUF1368 with unknown function specific to red algae.

Gfir_ORF1 and Gfir_ORF2 are two overlapping ORFs which appear to be truncated versions of single ORF (ORF5/ORF2) found in the Gch7220 or Gch3937 plasmids of *G. chilensis*, corresponding to amino acids 1 to 36 and 44 to 101, respectively (Additional file 1). Gfir_ORF20 which shares 76 and 74% amino acid identity with ORF5 from Gch7220 and ORF2 from Gch3937 at 199 amino acids is slightly longer than the comparable ORF from *G. chilensis* plasmids (Additional file 1). The high sequence identity may indicate recent horizontal transfer of this ORF from *G. chilensis*-specific plasmid to *G. firma* chloroplast. Gfir_ORF18 and Gfir_ORF19 display considerable amino acid similarity with ORF4 (61% amino acid identity corresponding to amino acids 17 to 91) and ORF7 (64% amino acid identity corresponding to amino acids 23 to 61) of Gro4970 plasmid respectively. The remaining ORFs are homologous to all five ORFs in the Gle4293 plasmid of *Gp. lemaneiformis*. Gfir_ORF17, Gfir_ORF16, Gfir_ORF15, Gfir_ORF14 and Gfir_ORF13 showed similarity to ORF1, ORF2, ORF3, ORF4 and ORF5 of Gle4293 plasmid (Fig. 4), with each ORF corresponding to amino acids 57 to 417 (53% identity), 16 to 132 (49% identity), 17 to 262 (53% identity), 1 to 168 (59% identity) and 17 to 149 (58% identity). Apart from Gfir_ORF17, Gfir_ORF12, Gfir_ORF21, Gfir_ORF22 and Gfir_ORF23 are also truncated versions of the ORF1 from Gle4293 plasmid, each corresponding to amino acids 1 to 161 (42% identity), 375 to 418 (59% identity), 275 to 359 (64% identity) and 226 to 274 (61% identity). The integration of whole plasmid into the chloroplast of *G. firma* may have occurred, considering the presence of degenerated fragments homologous to all five ORFs from Gle4293 plasmid in a contiguous manner in the intergenic region between the *nblA* and *cpeB* genes of the *G. firma* chloroplast genome.

Whole-genome alignment of the chloroplast genome of all five members of Gracilariales revealed a common syntenic break at the position between 30,000 and 40,000, which corresponds to the intergenic regions within the *rrs-ompR-psbD* cluster (Fig. 3). This region represents the “hotspot” for plasmid integration in the chloroplast genome of Gracilariales, as ORFs homologous to red algal plasmids were found in this region based on the previous studies [5, 6] (Fig. 4). The intergenic region between *ompR* and *psbD* in the chloroplast genome of *G. salicornia* has an ORF that shares strong similarity with ORF1 of Gch7220 plasmid [5, 6]. And the corresponding spacer region in the chloroplast genome of *G. chilensis* has a gene pair *leuC-leuD* of bacterial origin and three plasmid-derived regions which are homologous to the ORF1, ORF2 and ORF3 from Gle4293 plasmid respectively [6]. In the chloroplast genome of *G. tenuistipitata* var. *liui*, the intergenic region between *rrs* and *ompR* in the chloroplast genome was reported to have a plasmid-derived region homologous to ORF1 of Gle4293 plasmid [6], and the region between *ompR* and *psbD* has a gene pair *leuC-leuD* and an ORF similar to ORF14 of Gch7220 plasmid [5]. Three ORFs homologous to the ORF5 and ORF1 of Gle4392 plasmid were found in the spacer region between *rrs* and *ompR* in the chloroplast genome of *Gp. lemaneiformis*. A notable feature unique to the chloroplast genome of *G. firma* is the presence of a relatively large syntenic break with 12 red algal plasmid homologs spanning about 7 kb at position ~153,000 between *nblA* and *cpeB* (asterisk in Fig. 3). This region was not observed in any other examined taxa, and likely to have contributed to the expansion of chloroplast genome size in *G. firma*.

Despite the caveat that the sequenced library was not searched to confirm the existence of extrachromosomal plasmid in *G. firma*, we preclude the possibility of mapping artefact in the assembly for the expanded chloroplast genome on two grounds: (1) single contigs with identical sequence were obtained from two independent assemblies using different *de novo* and reference-guided assemblers, and (2) the presence of red algal plasmid remnants in the chloroplast genome has been reported in red algal taxa with or without naturally occurring extrachromosomal plasmid [4, 5, 38, 41, 42]. Previous studies have verified the occurrence of plasmid-derived regions in the chloroplast genome of *Gp. lemaneiformis* [5], and confirmed the consistency of the plasmid-derived sequences within individuals and populations of *Gelidium elegans*, *Porphyra pulchra* and *Sporolithon durum* [6], using customized primer pairs for PCR. The copy number and position of the homologous plasmid-derived ORFs in chloroplast genomes is inconsistent with the red algal phylogenetic relationships [6]. Incorporation of foreign genetic materials into the maternally inherited organelles may have rendered their fixation in a population. The plasmid may have recombined randomly during the integration into the chloroplast genome and led to gene truncation and subsequent gene loss, such that vestiges of red algal plasmid were perceived only in certain but not all red algal species. Such species-specific integration of plasmid-derived ORFs is consistent with the evolution of mobile genetic elements. The plasmids are thought to be analogous to transposable elements with mobility that can contribute to the gain or loss among closely related genomes [6]. However, the actual role of red algal plasmids remains elusive. While recognizing the possible role of red algal plasmids in mediating gene transfer between foreign DNA and organelles, a study considered the plasmids as parasitic elements that spread plasmid-derived DNA regions in different organelles [6]. It was suggested that the ubiquitous red algal plasmid remnants may be associated with their function in ancient horizontal gene transfer among nucleus, chloroplast and mitochondrion genomes [5].

### Evolution of chloroplast genome in Eurhodophytina

The gene content and gene order of 21 chloroplast genomes representing the classes Bangiophyceae and Florideophyceae was compared to infer the evolution of chloroplast genome in red algae. The highly divergent unicellular thermophiles of the classes Cyanidiophyceae and Porphyridiophyceae were not included in the genome alignment. Only one representative from each genus of Bangiophyceae was included in the analyses to emphasize the resolution of the relationship within Florideophyceae using chloroplast genome data. The parasitic red alga *Choreocolax* was also excluded from the genome alignment as it experienced many rearrangements and losses despite sharing regions of synteny with other Florideophycean taxa [43].

**Fig. 5:**
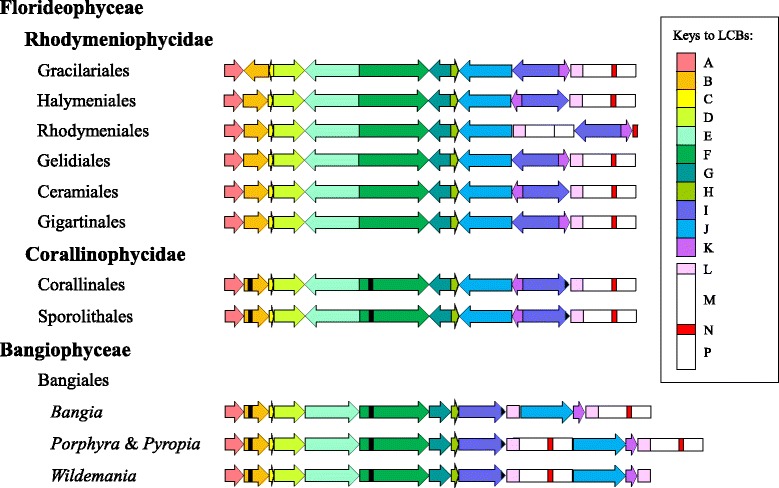
Schematic representation of the syntenic comparison for the chloroplast genomes of selected taxa across Eurhodophytina. Taxa are ordered as in the phylogeny in Fig. 6. Corresponding locally collinear blocks (LCBs) are depicted as color-coded arrow blocks (A–K) and rectangle blocks (L–P). Major syntenic differences between Florideophyceae and Bangiophyceae are due to inversion and translocation of the collinear blocks B, C, E, G, I, J, K and L. Blocks A–K encompass the conserved protein-coding genes and most of the tRNA genes, and the direction of arrowhead indicates only the orientation of the gene cluster in an LCB relative to the Bangiophyceae. *Black bars* denote the genes shared by Bangiophyceae and Corallinophycidae that are no longer present in Rhodymeniophycidae, including the *trnS* gene in block B, the *chlB* gene in block F, and the *chlL*-*chlN* gene pair in block J. Other lineage- or species-specific gene losses in Rhodymeniophycidae are not indicated. Blocks L–P represent the typical rRNA operon made up of the *rrf* (block L), *rrl* (block M), the two tRNA genes *trnA* and *trnI* (block N) and *rrs* genes (block P). The rRNA operon experienced translocation in Rhodymeniales, complete duplication as direct repeats in *Porphyra* and *Pyropia*, and partial duplication of the *rrf* gene in *Bangia* and *Wildemania*

A total of 229 chloroplast genes were inferred to present in the hypothetical last common ancestor of the Bangiophyceae and Florideophyceae, including 194 protein-coding genes, 3 rRNA genes, and 35 tRNA genes, based on the currently available chloroplast genomes examined in this study (Table 3). Comparison of the whole-genome alignments revealed a largely identical gene content in the chloroplast genome of Bangiophyceae and Florideophyceae with a few exceptions. The retention of such large number of chloroplast gene repertoires known in photosynthetic eukaryotes including the complete tRNA set and the genes encoding transcriptional regulators, and the position of genes on the same strand in operon-like structures, suggest ancient gene content in the red algal chloroplast genomes [38, 44]. Apart from the *glnB* gene flanked by the *rpl33* and *rps20* genes, the representatives of Bangiophyceae also featured three additional tRNA genes not found in the Florideophycean representatives, including a *trnA* gene in the tRNA gene cluster of *trnM-trnS-trnD*, a *trnS* gene positioned between *ftsH* and *psaE*, and a *trnL* gene between *fabH* and *psbX.* The *chlB*, *chlL* and *chlN* genes, along with the *trnS* gene positioned upstream of the *moeB* gene, which were initially considered exclusive to the Bangiophycean species [38] before more chloroplast genomes of Florideophyceae are available, were also found in the representatives of Corallinophycidae but not Rhodymeniophycidae (black bars in the arrow blocks B, F and J in Fig. 5). The absence of those genes encoding redundant tRNA variants and subunits of the chloroplast light-independent protochlorophyllide reductase was considered as outright gene loss rather than gene transfer from chloroplast to nucleus given that all the genes involved have been completely lost in many other photosynthetic eukaryotes [4]. Additional taxa sampling to include the other subclasses of Florideophyceae would reveal whether such pattern of gene loss is characteristic to the Florideophyceae. Nevertheless, the presence or absence of certain genes in the chloroplast genome should not be taken as reliable phylogenetic characters because gene transfer to the nucleus or gene loss has occurred numerous times independently across various eukaryotic lineages [45]. Lineage- or species-specific loss of a few genes including *dfr*, *dnaB*, *petL*, *pbsA*, *psb30*, *rpl9*, *syfB*, *syh*, *secG*, and *thiS* were also observed. A few instances are the loss of *dfr* in *Chondrus crispus*, *Vertebrata lanosa*, and *Laurencia snackeyi*, the independent loss of *syh*, *dnaB*, *syfB* and *ycf21* in *Sporolithon durum*, and loss of *pbsA* in *G. firma* and *G. chilensis*.

**Table 3:** Gene cluster content of Eurhodophytina

LCB	Conserved genes present in LCB
A	*orf243* [*ycf27* or *ompR*] *, psbD, psbC, orf198* [*upp*]*, ycf19, rps16, ycf65, groEL, trnR*(ACG)*, syh, trnQ*(UUG), *trnR*(CCU)
B	*psbW, syfB, rps1, orf71* [*ycf40* or *thiS*]*, trnH*(GUG)*, ycf29* [*tctD*]*, ycf28* [*ntcA*]*, petB, petD, rpl12, rpl1, rpl11, trnW*(CCA)*, orf277* [*moeB*]*, trnS*(GGA)*, rpl9*
C	*dnaB, trnF*(GAA)*, clpC*
D	*rpl19, trnV*(GAC)*, trnY*(GUA)*, trnT*(GGU)*, ilvB, ycf33, argB, trnM*(CAU)*, trnA*(GGC)*, trnS*(GCU)*, trnD*(GUC)*, ftsH, trnS*(CGA)*, psaE, psaH, psaN, psaT, psbB, ycf38, petF*
E	*rps14, petG, psbK, psbZ, trnG*(GCC)*, dnaK, rpl3, rpl4, rpl23, rpl2, rps19, rpl22, rps3, rpl16, rpl29, rps17, rpl14, rpl24, rpl5, rps8, rpl6, rpl18, rps5, secY, rpl36, rps13, rps11, rpoA, rpl13, rps9, rpl31, rps12, rps7, tufA, rps10*
F	*psaB, psaA, accB, orf565* [*ycf45*]*, acpP, trnS*(UGA)*, psaD, chlB, ycf59* [*ascF*]*, petN, orf71* [*secG* or *ycf47*]*, ycf36, trnM*(CAU)*, orf199* [*bas1*]*, pbsA, rpl35, rpl20, preA, odpA* [*pdhA*]*, odpB* [*pdhB*]*, petA, tatC, apcE, apcA, apcB, atpE, atpB, ycf3, infB, rps18, rpl33, rps20, rpoB, rpoC1, rpoC2, rps2, tsf, atpI, atpH, atpG, atpF, atpD, atpA, ycf16* [*sufC*]*, ycf24* [*sufB*]*, trnL*(CAA)*, ycf39, psbI, orf149* [*ycf58*]*, cemA, trnM*(CAU)
G	*infC, ilvH, trnL*(UAA)*, trnC*(GCA)*, cbbX, rbcS, rbcL, trxA, rpl28, trnT*(UGU)*, psaL, ycf7* [*petL*]*, ycf4, trnG*(UCC)
H	*rps4, orf450* [*ycf80*]*, trnR*(CCG)*, apcF, ycf20*
I	*trnL*(UAG) *, ycf35*, *psbA, rne, rpl27, rpl21, orf263* [*ycf56*]*, rpl32, ycf32* [*psbY*]*, rbcR, orf108* [*ycf54*]*, orf320* [*ycf55*]*, orf238* [*ycf53*]*, carA, petJ, psbV, accD, psbX, trnL*(GAG) *, fabH, apcD, psaJ, psaF, orf174* [*ycf52*]*, trnP*(UGG)*, ycf37*, *rpl34, ycf46, chlN, chlL*
J	*psaM, chlI, trnR*(UCU)*, trnV*(UAC)*, orf263a* [*ycf63*]*, ycf26* [*dfr*]*, psbE, psbF, psbL, psbJ, psaI, ftrB, ycf12* [*psb30*]*, orf327* [*ycf62* or *tilS*]*, trnK*(UUU)*, trpA, trnE*(UUC)*, secA, ycf21, pgmA, cpcA, cpcB, ycf61* [*rpoZ*]*, gltB, psaC, ycf23, ycf22, accA, psaK, ccsA, cpeA, cpeB, ycf18* [*nblA*]*, cpcG*
K	*trnN*(GUU)*, orf240* [*dsbD* or *ccdA*]*, ccs1, trpG, thiG, orf203* [*ycf60*]*, rps6*
L	*Rrf*
M	*Rrl*
N	*trnA*(UGC), *trnI*(GAC)
P	*rrs*

The gene content inferred to present in the hypothetical last common ancestor of Bangiophyceae and Florideophyceae are listed in conserved clusters identified using Mauve. The alphabetical code of each conserved gene cluster or locally collinear block (LCB) corresponds to that in Fig. 5. The genes in each cluster are listed in the order they present in the Bangiophyceae in Fig. 5. Alternative names applied for the homologous genes in the Florideophyceae are indicated in square brackets

Fifteen LCBs were identified chloroplast genomes, with blocks A–K comprising the protein-coding and tRNA genes, and blocks L–P representing the rRNA operon (Fig. 5). Blocks E, F, I and J encompassed a large portion of the conserved gene repertoire of red algal chloroplast genomes. Some blocks comprised of only one or two genes, including blocks L, M, N and P, as a result of single gene translocation (Table 3). Most of the genomic rearrangements involved simultaneous inversion or translocation of gene cluster in the collinear blocks B, C, E, G, I, J, and K. Major syntenic differences observed between Bangiophyceae and Corallinophycidae were the inversion of collinear blocks E, G, J and K, and the translocation of block I from the original position immediately downstream of block H exhibited by Bangiophyceae, to a novel position between block K and the rRNA operon. The gene order has been highly conserved since the split from Bangiophyceae and a high degree of synteny was observed in Florideophyceae. Representatives of the Corallinophycidae, Ceramiales and Halymeniales exhibited similar gene order across the examined Florideophycean lineages, with some gene losses in the Rhodymeniophycidae as mentioned above. An inversion of the collinear block (I + K) occurred in the Gracilariales, Gigartinales, Gelidiales and Rhodymeniales with respect to its orientation in the Corallinophycidae, Ceramiales and Halymeniales. In addition, *C. compressa* of the Rhodymeniales featured a unique translocation of the three rRNA genes (blocks L, M and P) from the original position downstream of block K as in the Gracilariales, Gigartinales and Gelidiales, to a position flanked by block J upstream and block I downstream. The Gracilariales exclusively exhibited inversion of the blocks B and C not seen in other orders.

The Florideophycean taxa possessed only a single copy of rRNA operon typically comprised of the *rrf*, *rrl*, *trnA*, *trnI* and *rrs* genes, except for the rRNA operon of *Coeloseira compressa* which comprised only three rRNA genes. The representatives of Bangiophyceae showed more variations in the number of the rRNA genes. *Porphyra umbilicalis* and *Pyropia haitanensis* possessed two copies of rRNA operon each made up of the *rrf*-*rrl*-*trnA*-*trnI*-*rrs* gene cluster as direct repeats, with one copy of the operon positioned between blocks I and J, and another downstream of block K. Hughey et al. [46] reported that *Pyropia perforata* lacked the second copy of rRNA operon found in other species of *Porphyra* and *Pyropia*. Instead of having two copies of rRNA operon like most species of *Porphyra* and *Pyropia,* both *Bangia atropurpurea* and *Wildemania schizophylla* have an additional *rrf* gene on top of the single copy of rRNA operon possessed.

Inclusion of more red algal taxa of the Bangiophyceae and Florideophyceae for the comparison of genomic synteny has resulted in the observation of more rearrangement patterns across the subphylum Eurhodophytina, with an increase in the number of conserved gene cluster from 11 in previous studies [4, 38] to 15 in present study. The chloroplast genomes of the Bangiales which had four genera examined in the present study exhibited identical genome structure when the rRNA operons were not taken into consideration. The red algal chloroplast genomes are highly conserved in gene content and order, considering the relatively minimal number of gene lost and extent of genomic rearrangement across Eurhodophytina over a substantial evolutionary distance since the divergence of Bangiophyceae and Florideophyceae that has occurred at least 940 million years ago [5, 47]. However, the gene order pattern was not reflective of the ordinal relationships inferred from the phylogenomic analyses. Parallel evolution in gene order is observed in Florideophyceae such that identical gene order pattern arose independently several times in different lineages.

### Phylogenetic relationships inferred using the chloroplast genome

**Fig. 6:**
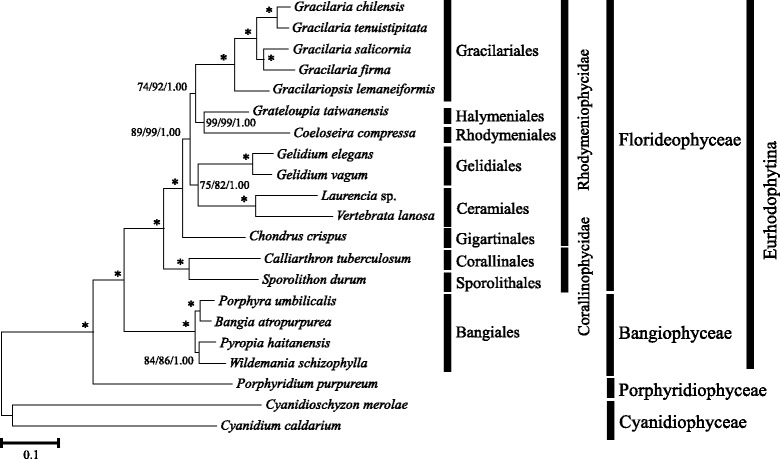
Phylogenetic tree of Eurhodophytina. Maximum-likelihood (ML) phylogeny inferred from the concatenated amino acid dataset of 146 plastid protein-coding genes for 21 taxa. Branch support measures are expressed as ML bootstrap percentages, SH-aLRT values and Bayesian posterior probabilities (from left to right). Asterisk denotes maximum nodal support

The phylogenies inferred using different phylogenetic approaches (ML vs BI) and gene sampling (79 vs 146 concatenated protein-coding genes) on 21 taxa recovered topologies which are largely congruent, with the exception in the Bayesian phylogeny inferred using the 79-gene dataset, where *Coeloseira compressa* was positioned basal to the Ceramiales. Additional gene sampling improved the branch support for the relationships between Gelidiales and Ceramiales with the SH-aLRT value increased from 40 in the 79-gene dataset to 82 in the 146-gene dataset. Only the maximum likelihood (ML) phylogeny inferred from the concatenated dataset containing 146 protein-coding genes of the chloroplast genomes present in all 21 examined taxa was presented (Fig. 6), with the posterior probabilities recovered from the Bayesian analysis appended on the nodes of the ML phylogeny along with the SH-aLRT and bootstrap branch supports. Consistent with previous studies [47–49], Fig. 6 resolved the subphylum Eurhodophytina as two fully supported monophyletic groups corresponding to the classes Bangiophyceae and Florideophyceae. The subclass Corallinophycidae represented by *Calliarthron tuberculosum* (Corallinales) and *Sporolithon durum* (Sporolithales) was positioned basal to the subclass Rhodymeniophycidae with maximum nodal support. *Chondrus crispus* (Gigartinales) diverged first within the subclass Rhodymeniophycidae with strong nodal support (BS = 89%, SH-aLRT = 99; PP = 1.00). The remaining taxa of Rhodymeniophycidae were split into two groups, of which the basal group encompassed the Gelidiales (*Gelidium*) and Ceramiales (*Vertebrata lanosa* and *Laurencia snackeyi*), and another group consisted of Gracilariales (*Gracilaria salicornia, G. firma, G. chilensis, G. tenuistipitata* var. *liui* and *Gracilariopsis lemaneiformis*), Halymeniales (*Grateloupia taiwanensis*) and Rhodymeniales (*Coeloseira compressa*). The interordinal relationships within the Rhodymeniophycidae received moderate to strong nodal support (BS > 70%; SH-aLRT > 80; PP = 1.00). The relationships within the monotypic family of Gracilariales were fully supported with *Gp. lemaneiformis* being positioned basal to the remaining *Gracilaria* species.

The relationships among most of the orders in the subclass Rhodymeniophycidae have been identified as one of the evolutionary lineages in the red algal phylogeny by Verbruggen et al. [50]. These relationships require considerable analyses by the inclusion of more markers and wider taxon sampling to increase the informative characters for better phylogenetic resolution. The poorly supported interordinal relationships within the subclass is largely attributed to the rapid radiation of lineages (i.e. the Gigartinales *sensu lato*) at the base of the subclass. We refrain from drawing concluding remarks on the phylogenetic relationships of the Rhodymeniophycidae with notion that the mere addition of one taxon from previously studied Gracilariales in the underrepresented taxon sampling will not result in significant changes to the interordinal relationships within the subclass. Relative phylogenetic affinities of the taxa (or the representative orders) examined in this study based on the analyses using chloroplast genome data in this study were compared with those attained from previous work based on different sets of markers over wide sampling [47–50]. Interordinal relationships within the subclass Rhodymeniophycidae recovered in this study are congruent with the results inferred using the mitochondrial genome [48] and also multiple markers of different genomic origins in some studies (*rbcL*, *psaA*, *psbA*, EF2, SSU, LSU and *cox1* [47]; SSU, LSU, EF2, *rbcL* and CO1-5P [49]). These results, however, are in contrast with that inferred using DNA data of 14 markers (EF2, 23S rDNA, 28S rDNA, 16S rDNA, 18S rDNA, *cox1*, *psaA*, *psaB*, *psbA*, *psbC*, *psbD*, *rbcL*, *rbcS* and *tufA*) belonging to all three genomic compartments mainly mined from GenBank, of which ten markers were poorly represented [50]. The first divergence of *Chondrus crispus* in Gigartinales from other orders received strong nodal support in this study and moderate to strong support in previous studies [47–49]. The placement of the family accommodating this taxon in the most recently derived order in Florideophyceae was poorly supported in [50]. In contrast to the poorly supported grouping of Gracilariales with Ceramiales and Gelidiales in [50], this study recovered a moderately supported relationships among the Gracilariales, Halymeniales and Rhodymeniales (BS = 74, PP = 1.00). Consistent relationships among Gracilariales, Halymeniales and Rhodymeniales with moderate nodal support (BS = 67, PP = 1.00) was also recovered in the phylogeny inferred using mitochondrial genome and wider taxon sampling [48], but the relationships among these three orders were poorly supported in phylogenies constructed using multigene data over broad taxonomic breadth of Rhodymeniophycidae [47, 49]. While large multi-locus datasets for a broad taxonomic breadth are considered to be the preferred solution to resolve the relationships in the Rhodymeniophycidae [50], genome-scale data based on limited taxon sampling as in this study can still recover similar phylogenetic inference for the common taxa used in different studies. This suggests that the use of chloroplast genome data could help to improve the support for the interordinal relationships previously identified using multiple markers on Florideophyceae-wide sampling.

Classification in the family Gracilariales has always been in a state of flux over the years, with more than hundred species passing under the generic names of *Gracilaria* and *Hydropuntia* based on the morphological and anatomical features [14–16]. It was not until the rather comprehensive molecular study based on the *rbcL* gene had successfully delineated the *Gracilaria sensu lato* into three groups which correspond to a new lineage and the previously defined genera of *Gracilaria* and *Hydropuntia*, that the systematics within Gracilariaceae was considered stabilized [14]. *Hydropuntia* was maintained as a genus distinct from *Gracilaria* based on the well-circumscribed reproductive features, despite the lineage being supported by high Bayesian posterior probabilities and low ML bootstrap percentages in the molecular phylogeny [14]. It differs from the *Gracilaria sensu stricto* which features cystocarps composed of gonimoblasts and carposporangia arranged in longer chains that are dichotomously or irregularly branching and tubular filaments connecting the gonimoblasts to the pericarp, by possessing cystocarps composed of gonimoblasts cells in short chains terminating in apical carposporangia that are arranged radially, and tubular filaments connecting the gonimoblasts to the base of the cystocarp. However, a recent study on the phylogeny of Gracilariaceae using the *rbcL*, *cox1* and UPA indicated the non-monophyly of *Hydropuntia* and suggested its reduction to *Gracilaria* [16]. The taxa of Gracilariaceae examined in present study are representative of the three previously delineated groups, with *G. salicornia* as *Gracilaria sensu stricto* [14], *G. firma* as *Hydropuntia* [51], and *G. tenuistipitata* and *G. chilensis* as the new lineage [14]. Despite the limited taxon sampling, the fully resolved phylogeny of Gracilariaceae inferred using the concatenated dataset of 146 chloroplast protein-coding genes in this study implied the potential of chloroplast genome in resolving the *Gracilaria sensu stricto* conundrum. Inclusion of more samples of *Gracilaria sensu lato* for analyses may eventually lead to the recognition of *Hydropuntia* as a genus distinct from *Gracilaria sensu stricto*.

The interpretation and accuracy of the branch support measured as ML bootstrap replicate proportions and Bayesian posterior probabilities is still an issue of dispute, especially when the deep relationships involving rapid radiation of lineages are considered, although the likelihood-based phylogenetic reconstruction in the non-parametric maximum-likelihood and Bayesian frameworks have established themselves as the methods of choice [52]. The very short internal branches separating the major lineages of Gracilariales, Halymeniales, Rhodymeniales, Gelidiales and Ceramiales with long terminal branches (Fig. 6) suggest that these lineages of the subclass Rhodymeniophycidae underwent a rapid radiation [50]. The branching order is difficult to resolve when there is a rapid accumulation of many lineages in short period of time and when sequences become saturated, where the phylogenies typically exhibit very short internal branches with low ML bootstrap but high Bayesian posterior probabilities supports. It has been noted that the ML bootstrapping is computational intensive and often underestimates the branch support, whereas the Bayesian estimators inflate the confidence of the corresponding branches [53]. The values estimated by SH-aLRT, an alternative non-parametric approach to the conventional bootstrapping, which corroborated most of the relationships supported by high Bayesian posterior probabilities in this study, are likely to be another measure to support the data accurately. The branch supports derived from SH-aLRT are considered to be more consistent and conservative than conventional bootstrapping [54]. In addition, Bayesian posterior probabilities may be more indicative of the evolutionary relationships compared to the ML bootstrap replicate proportions which have been typically considered conservative when the full alignment was analyzed, as the deep relationships received increased ML bootstrap supports parallel to the high Bayesian posterior probabilities when analyses were conducted on the progressively more conservative alignments generated by site-stripping [49].

## Conclusions

This study presents the chloroplast genome of *Gracilaria firma* and identified unique red algal plasmid DNA remnants in the genome. Despite some lineage- and species-specific gene losses in the Florideophyceae, the chloroplast genomes across the subphylum Eurhodophytina are highly conserved in synteny and genome architecture. The chloroplast genomes hold substantial information that can be tapped for improving the resolution of the phylogenetic relationships at all taxonomic hierarchical levels. However, additional study including improved taxon sampling, additional sequence data and further exploration of analyses options such as data partitions and evolutionary model selections is warranted to resolve the relationships within the Rhodymeniophycidae [47–50].

## Additional file

Additional file 1: Unique ORFs of *Gracilaria firma* homologous to red algal plasmids. The *G. firma* ORFs showed significant similarity (*e*-value < 1e-5) to the plasmids of *Gracilariopsis lemaneiformis*, *G.chilensis* and *G. robusta*, with the percentage identity and similarity significance expressed after the plasmid homolog. (DOCX 14 kb)
