# Supplementary material for: Complete chloroplast genome of Gracilaria firma (Gracilariaceae, Rhodophyta), with discussion on the use of chloroplast phylogenomics in the subclass Rhodymeniophycidae
Source: BMC Genomics. 2017 Jan 6;18:40. doi: 10.1186/s12864-016-3453-0 (PMC5217408; doi:10.1186/s12864-016-3453-0)
Supplement: Additional file 1: — Unique ORFs of Gracilaria firma homologous to red algal plasmids. The G. firma ORFs showed significant similarity (e-value < 1e-5) to the plasmids of Gracilariopsis lemaneiformis, G.chilensis and G. robusta, with the percentage identity and similarity significance expressed after the plasmid homolog. (DOCX 14 kb) [file 12864_2016_3453_MOESM1_ESM.docx]

**Table S1 Unique ORFs of *Gracilaria* *firma* homologous to red algal plasmids.** The *G. firma* ORFs showed significant similarity (e-value<1e-5) to the plasmids of *Gracilariopsis lemaneiformis*, *G.chilensis* and *G. robusta*. Each plasmid homolog provided with accession number is followed by percentage identity and similarity significance.

| ORF | ORF size (bp) | Homologous red algal plasmid ORF | | | |
| --- | --- | --- | --- | --- | --- |
|  |  | Gle4293 of *Gracilariopsis lemaneiformis* | Gch7220 of *Gracilaria chilensis* | Gch3937 of *Gracilaria chilensis* | Gro4970 of *Gracilaria robusta* |
| Gfir_ORF2 | 237 |  | ORF5 (NP_052218), 63%, 2e-11 | ORF2 (NP_052236), 64%, 6e-6 |  |
| Gfir_ORF3 | 177 |  | ORF5 (NP_052218), 63%, 2e-11 | ORF2 (NP_052236), 64%, 6e-6 |  |
| Gfir_ORF12 | 477 | ORF1 (NP_052230), 42%, 1e-22 | ORF1 (NP_052214), 47%, 1e-11;  ORF4 (NP_052217), 36%, 5e-11 | ORF1 (NP_052235), 36%, 2e-10 | ORF1 (NP_052207), 38%, 1e-8 |
| Gfir_ORF13 | 410 | ORF5 (NP_052234), 58%, 1e-51 |  |  |  |
| Gfir_ORF14 | 687 | ORF4 (NP_052233), 59%, 5e-61 |  |  |  |
| Gfir_ORF15 | 780 | ORF3 (NP_052232), 53%, 9e-85 |  |  |  |
| Gfir_ORF16 | 519 | ORF2 (NP_052231), 49%, 1e-28 |  |  |  |
| Gfir_ORF17 | 1077 | ORF1 (NP_052230), 53%, 3e-126 | ORF4 (NP_052217), 34%, 2e-42;  ORF6 (NP_052220), 41%, 8e-11 | ORF1 (NP_052235), 34%, 6e-43 | ORF1 (NP_052207), 32%, 3e-33 |
| Gfir_ORF18 | 426 |  |  |  | ORF4 (NP_052210), 61%, 3e-20 |
| Gfir_ORF19 | 207 |  |  |  | ORF7 (NP_052212), 64%, 6e-6 |
| Gfir_ORF20 | 597 |  | ORF5 (NP_052218), 76%, 4e-108 | ORF2 (NP_052236), 74%, 5e-91 |  |
| Gfir_ORF21 | 126 | ORF1 (NP_052230), 59%, 2e-6 |  |  |  |
| Gfir_ORF22 | 339 | ORF1 (NP_052230), 64%, 6e-27 | ORF4 (NP_052217), 39%, 1e-9;  ORF6 (NP_052220), 40%, 8e-10 | ORF1 (NP_052235), 39%, 1e-9 | ORF1 (NP_052207), 45%, 1e-10 |
| Gfir_ORF23 | 162 | ORF1 (NP_052230), 61%, 8e-11 |  |  |  |
